# Postextubation laryngeal edema and stridor resulting in respiratory failure in critically ill adult patients: updated review

**DOI:** 10.1186/s13054-015-1018-2

**Published:** 2015-09-23

**Authors:** Wouter A. Pluijms, Walther NKA van Mook, Bastiaan HJ Wittekamp, Dennis CJJ Bergmans

**Affiliations:** Department of Anesthesiology, Zuyderland Medical Centre, Henri Dunantstraat 5, Postbus 4446, 6401 CX, Heerlen, The Netherlands; Department of Intensive Care Medicine, Maastricht University Medical Centre, P. Debyelaan 25, Postbus 5600, 6202 AZ Maastricht, The Netherlands; Julius Center for Health Sciences and Primary Care, University Medical Centre Utrecht, Heidelberglaan 100, Postbus 85500, 3584 CX Utrecht, The Netherlands

## Abstract

Endotracheal intubation is frequently complicated by laryngeal edema, which may present as postextubation stridor or respiratory difficulty or both. Ultimately, postextubation laryngeal edema may result in respiratory failure with subsequent reintubation. Risk factors for postextubation laryngeal edema include female gender, large tube size, and prolonged intubation. Although patients at low risk for postextubation respiratory insufficiency due to laryngeal edema can be identified by the cuff leak test or laryngeal ultrasound, no reliable test for the identification of high-risk patients is currently available. If applied in a timely manner, intravenous or nebulized corticosteroids can prevent postextubation laryngeal edema; however, the inability to identify high-risk patients prevents the targeted pretreatment of these patients. Therefore, the decision to start corticosteroids should be made on an individual basis and on the basis of the outcome of the cuff leak test and additional risk factors. The preferential treatment of postextubation laryngeal edema consists of intravenous or nebulized corticosteroids combined with nebulized epinephrine, although no data on the optimal treatment algorithm are available. In the presence of respiratory failure, reintubation should be performed without delay. Application of noninvasive ventilation or inhalation of a helium/oxygen mixture is not indicated since it does not improve outcome and increases the delay to intubation.

## Introduction

Laryngeal edema (LE) is a frequent complication of intubation and is caused by trauma to the larynx [1, 2]. The edema results in a decreased size of the laryngeal lumen, which may present as stridor or respiratory distress (or both) following extubation. Ultimately, postextubation laryngeal edema (PLE) may lead to respiratory failure with subsequent need for reintubation. Since reintubation is associated with increased morbidity and mortality, it is important to prevent reintubation if possible [3]. Recent studies have focused on several methods to assess airway patency before extubation, aiming to identify patients at risk for PLE. This may enable timely and targeted treatment of patients at risk for postextubation respiratory failure (PRF). This review provides an update on this topic, focusing on these recent developments [4].

## Etiology

PRF may result from liberation failure (i.e., the inability to ventilate spontaneously without ventilator support) or extubation failure (i.e., the inability to tolerate removal of the endotracheal tube) or both [5]. Liberation failure may result from primary respiratory failure, congestive heart failure, or neurological impairment. Causes of extubation failure include upper airway obstruction and inadequate clearance of airway secretions [5, 6].

Endotracheal intubation causes damage to the airway in most patients, leading to LE, ulcerations, and damage to the vocal cords [1, 7–9]. Although these injuries are generally reversible, they may cause a decrease of the available airway lumen and lead to respiratory difficulty directly after extubation [1, 7, 9]. The decreased airway lumen results in an increase of air flow velocity, leading to postextubation stridor (PES), which is a clinical marker of relevant PLE. Although the exact quantitative relationship between lumen narrowing and clinical symptoms is unclear, the presence of respiratory distress and PES is thought to reflect a narrowing of the airway lumen of more than 50 % [10].

## Incidence of postextubation laryngeal edema, stridor, and respiratory failure

### Postextubation laryngeal edema and stridor

Earlier studies have reported an incidence of PLE ranging from 5.0 % to 54.4 % (Table 1) [2, 11–15]. The large variation may be explained by the lack of a standardized method to identify LE, resulting in the use of different definitions of PLE in various studies. Similarly, the reported incidence of PES varies widely; estimated incidence ranges from 1.5 % to 26.3 % [1, 14–29].

**Table 1:** Incidence of postextubation stridor, postextubation laryngeal edema, and reintubation

Author	Year	Participants, number	Cases, number	Percentage	Reintubation due to PES/PLE, number	Percentage of participants	Percentage of cases	Reintubation total, number	Percentage of participants
Postextubation stridor									
Colice et al. [1]	1989	82	5	6.1 %	N/A	N/A	N/A	9	11 %
Ho et al. [17]^a^	1996	38	10	26.3 %	1	2.6 %	10 %	N/A	N/A
Miller and Cole [22]	1996	100	6	6.0 %	3	3.0 %	50 %	17	17.0 %
Epstein and Ciubotaru [3]	1998	745	N/A	N/A	11	1.5 %	N/A	74	9.9 %
Sandhu et al. [21]	2000	110	13	11.8 %	6	5.5 %	46.2 %	N/A	N/A
De Bast et al. [2]	2002	76	10	13.2 %	8	10.5 %	80 %	14	18.4 %
Jaber et al. [25]^b^	2003	112	13	11.6 %	9	8.0 %	69.2 %	11	9.8 %
Maury et al. [20]^b^	2004	99	4	4.0 %	1	1.0 %	25.0 %	18	18.2 %
Kriner et al. [18]	2005	462	20	4.3 %	7	1.5 %	35 %	N/A	N/A
Ding et al. [23]	2006	51	4	7.8 %	2	3.9 %	50 %	N/A	N/A
Cheng et al. [16]^c^	2006	236	18	7.6 %	10	4.2 %	55.6 %	14	5.9 %
Lee et al. [30]^d^	2007	325	25	7.7 %	6	1.8 %	24.0 %	6	1.8 %
Tadié et al. [14]	2010	136	18	13.2 %	4	2.9 %	22.2 %	17	12.5 %
Cheng et al. [26]^c^	2011	113	16	14.2 %	11	9.7 %	68.8 %	14	12.4 %
Gros et al. [27]	2012	104	7	6.7 %	6	5.8 %	85.7 %	29	27.9 %
Sutherasan et al. [15]	2013	101	16	15.8 %	N/A	N/A	N/A	N/A	N/A
Mikaeili et al. [28]	2014	41	4	9.8 %	N/A	N/A	N/A	N/A	N/A
Abbasi et al. [29]^a^	2014	35	7	20 %	N/A	N/A	N/A	11	31.4 %
Laryngeal edema									
Darmon et al. [12]^a^	1992	337	17	5.0 %	5	1.5 %	29.4 %	N/A	N/A
De Bast et al. [2]	2002	76	8	10.5 %	8	10.5 %	100 %	14	18.4 %
Chung et al. [13]	2006	95	35	36.8 %	N/A	N/A	N/A	N/A	N/A
François et al. [11]^a^	2007	343	76	22.2 %	11	3.2 %	14.5 %	26	7.6 %
Tadié et al. [14]	2010	136	74	54.4 %	13	9.6 %	17.6 %	N/A	N/A
Sutherasan et al. [15]	2013	101	17	16.8 %	2	2.0 %	11.8 %	N/A	N/A

Adapted from Wittekamp et al. [4]

*PES/PLE* postextubation stridor/postextubation laryngeal edema, *N/A* data not available

^a^Placebo group

^b^Only reintubation after first extubation attempt was included in analysis

^c^Nonintervention group (cuff leak volume (CLV) ≥24 %) and placebo group combined

^d^Nonintervention group (CLV >110 ml) and placebo group combined

### Postextubation respiratory failure

As mentioned earlier, PRF may result from liberation failure or extubation failure [5]. The reported overall incidence of PRF leading to reintubation ranges from 1.8 % to 31.4 % (Table 1) [1–3, 11–14, 16–18, 20–23, 25–30]. The reported incidence of reintubation due to PLE or PES (or both) is 1.1–10.5 %, whereas reintubation is necessary in 10.0–100 % of patients with PES or PLE or both. Given the available data, it is unclear what percentage of PRF is caused by PLE, although from the available evidence PLE and PES seem to be important contributors to the overall incidence of PRF.

## Risk factors

Several studies have aimed to identify risk factors for PLE and PES (Table 2) [1, 3, 7, 11, 12, 16–21, 25, 31–33]. Important risk factors include female gender, longer duration of intubation, use of large tube size and high cuff pressure, and difficult intubation. Unfortunately, none of these risk factors is sufficiently reliable to identify patients at risk for PLE and this prevents targeted treatment of high-risk patients.

**Table 2:** Risk factors for complications following extubation

Outcome measure	Study	Year	Risk factors
Laryngeal injury	Colice et al. [1]	1989	Persistent laryngeal neuromotor activity, tracheostomy
	Kastanos et al. [7]	1983	Severe respiratory failure, high cuff pressure, duration of endotracheal intubation, secretion infection
	Esteller-Moré et al. [31]	2005	Longer duration of intubation, tracheostomy, number of days in the intensive care unit
Laryngeal edema	Darmon et al. [12]	1992	Duration of intubation (>36 hours, female gender)
	François et al. [11]	2007	Trauma at admission, gender (female), short duration of intubation (<7 days), smaller height-to-tube diameter ratio, absence of methylprednisolone pretreatment
Postextubation stridor	Cheng et al. [16]	2006	Gender (female), lower Glasgow Coma Scale score, non-sedation treatment
	Sandhu et al. [21]	2000	Duration of intubation (>3 days)
	Daley et al. [32]	1996	Tracheostomy, time to reintubation
	Ho et al. [17]	1996	Gender (female)
	Jaber et al. [25]	2003	High SAPS II, medical patients, difficult intubation, history of self-extubation, prolonged intubation, high cuff pressure
	Kriner et al. [18]	2005	Gender (female), duration on intubation (>6 days), ration tube size to laryngeal size >45 %
	Wang et al. [19]	2007	Gender (female)
	Maury et al. [20]	2004	Gender (female)
	Erginel et al. [33]	2005	Duration of ventilation (>5 days), body mass index (>26.5)
Reintubation	Daley et al. [32]	1996	Tracheostomy, postextubation stidor
	Jaber et al. [25]	2003	Postextubation stridor
	Epstein and Ciubotaru [3]	1998	APACHE II score, age, cardiopulmonary cause for reintubation
	Sandhu et al. [21]	2000	Duration of previous intubation (>3 days)

Adapted from Wittekamp et al. [4]

*SAPS II* Simplified Acute Physiology Score II, *APACHE II* Acute Physiology and Chronic Health Evaluation II

## Assessment of airway patency before extubation

In an effort to allow identification of patients at risk for PLE, several tests have been evaluated for the assessment of airway patency before extubation, including the cuff leak test (CLT), ultrasonography, and video laryngoscopy.

### Cuff leak test

The CLT is an easy-to-perform, non-invasive test which provides information on the available laryngeal lumen and has been evaluated in several studies (Tables 3 and 4) [2, 13, 16, 18–22, 25, 27, 28, 34, 35]. The difference between the inspiratory tidal volume and the averaged expiratory tidal volume with the balloon deflated is defined as the cuff leak volume (CLV). The CLV is then compared with a predefined cutoff value, yielding a negative (CLV ≥ cutoff value) or positive (CLV < cutoff value) result. Whereas the positive predictive value for PES strongly differs according to the used cutoff value, the negative predictive value is consistently above 90 % in the studies addressing this test. In a recent study, the results of a CLT before extubation were compared with the results of a CLT performed directly after intubation [27]. Given that no LE is present at intubation, the difference (ΔCLT) reflects the decrease of available airway lumen caused by LE [27]. With a cutoff value of 0 ml, indicating an absence of LE, sensitivity (86 %), specificity (48 %), positive predictive value (11 %), and negative predictive value (99 %) were calculated and they were not superior to those of conventional CLT. Therefore, the CLT is mainly effective in identifying patients not at risk for PLE or PES.

**Table 3:** Measurement of the cuff leak volume in mechanically ventilated patients

Before performing the cuff leak test, first suction endotracheal and oral secretions and set the ventilator in the assist control mode.
With the cuff inflated, record displayed inspiratory and expiratory tidal volumes to see whether these are similar. Record cuff pressure.
Deflate the cuff.
Directly record the expiratory tidal volume over the next six breathing cycles as the expiratory tidal volume will reach a plateau value after a few cycles.
Average the three lowest values.
The difference between the inspiratory tidal volume (measured before the cuff was deflated) and the averaged expiratory tidal volume is the cuff leak volume.

Reprinted with permission from Wittekamp et al. [4]. Edited from Miller and Cole [22]

**Table 4:** Predictive value of the cuff leak test and laryngeal ultrasonography for postextubation stridor, laryngeal edema, and reintubation

Author	Year	Predefined cutoff value		Outcome	Sensitivity	Specificity	PPV	NPV
Cuff leak test		Volume, ml	Percentage of tidal volume					
Miller and Cole [22]	1996	110		Stridor	0.67 (0.51–0.82)	0.99	0.80	0.98
Jaber et al. [25]	2003	130	12	Stridor	0.85 (0.65–0.99)	0.95 (0.91–0.99)	0.69	0.98
De Bast et al. [2]	2002		15.5	Reintubation	0.75	0.72	0.25	0.96
Sandhu et al. [21]	2000		10.0	Stridor or reintubation	0.54	0.96	0.64	0.94
Wang et al. [19]	2007	88		Stridor	0.60	0.89	0.55	0.91
Maury et al. [20]	2004	0		Stridor	1.00	0.80	0.15	1.00
Chung et al. [13]	2006	140		Laryngeal edema	0.89	0.90	0.84	0.93
Engoren [34]	1999	110		Stridor	0.00	0.96	0.00	0.99
Kriner et al. [18]	2005	110		Stridor	0.50	0.84	0.12	0.97
Cheng et al. [16]	2006		18.0	Stridor	0.85	0.72	0.21	0.98
Mikaeili et al. [28]	2014	110		Stridor	0.25	0.84	0.14	0.91
		130			0.25	0.81	0.13	0.91
		249			0.75	0.59	0.17	0.96
Gros et al. [27]	2012	130		Stridor	0.86 (0.42–1.00)	0.76 (0.67–0.84)	0.21 (0.08–0.40)	0.99 (0.93–1.00)
Sutherasan et al. [15]	2013	110		Laryngeal edema	0.80	0.82	0.46	0.96
Ultrasonography		ACWD, mm						
Sutherasan et al. [15]	2013	1.6		Laryngeal edema	0.71	0.70	0.32	0.92
Mikaeili et al. [28]	2014	0.85		Stridor	0.50	0.57	0.11	0.91

95 % confidence intervals are provided if available

Adapted from Wittekamp et al. [4]

*PPV* positive predictive value, *NPV* negative predictive value, *ACWD* air column width difference

The poor performance of the CLT in the identification of high-risk patients might be explained in part by the relatively low prevalence of PLE and PES in most studies on the predictive value of the CLT. However, the low positive predictive value of the CLT for PLE and PES might be explained by the characteristics of the test as well and this has been shown in a physiological study [36]. For an optimal positive predictive value of the CLT, the cross-sectional area around the deflated cuff should be the only determinant of the CLV. However, this study indicated that the outcome of the CLT is significantly affected by the inspiratory leak volume since the expiratory leak volume is only 30 % of the total leak volume. This inspiratory leak volume is significantly affected by the respiratory system compliance and inspiratory flow, which are therefore additional determinants of the CLV. Therefore, the timing of cuff deflation is important, and ideally the cuff should be deflated immediately prior to expiration to eliminate the inspiratory leak volume. However, to the best of our knowledge, no published studies have compared the conventional CLT with an adapted version of the CLT, in which the cuff is deflated only during expiration.

### Ultrasonography

Several studies on the assessment of airway patency using ultrasonography have been published in recent years [15, 23, 28]. Using ultrasonography, the air column width (ACW), which is defined as the width of the acoustic shadow present at the level of the vocal cords, can be measured. If the ACW is measured before and after endotracheal cuff deflation, the air column width difference (ACWD) can be calculated. Results from the study by Ding et al. showed a significantly lower ACWD (0.35 versus 1.5 mm; *P* < 0.01) and lower ACW during cuff deflation (4.5 versus 6.4 mm; *P* = 0.01) in patients who developed a stridor compared with patients who did not [23]. Owing to the small sample size (*n* = 51), including only four patients with PES, results from this study should be interpreted with caution. In the study by Sutherasan et al., similar results were found with decreased ACW after cuff deflation (5.97 versus 6.87 mm; *P* < 0.05) and ACWD (1.08 versus 1.99 mm; *P* < 0.001) in patients who developed PLE [15]. Unfortunately, these results could not be replicated in the study by Mikaeili et al. [28]. They reported no significant difference regarding ACW before deflation (12 versus 11.5 mm; *P* = 0.48) or ACWD (0.1 versus 1.0 mm; *P* = 0.59) between patients with and without PES. This might be explained by the small sample size (*n* = 41) and the subsequent small number of patients developing PES (*n* = 4). Further statistical analysis of available evidence indicates that ultrasonography has a low positive predictive value, sensitivity, and specificity for predicting PES or PLE or both (Table 4). However, these findings should be interpreted with caution since available evidence is limited to small-scale studies with only small numbers of patients with PES or PLE or both.

### Video laryngoscopy

Results on the use of video laryngoscopy for the assessment of airway patency have been published in one case series, including only three patients [37]. The case series included two patients with PES and one patient with LE secondary to extensive airway manipulation due to an unanticipated difficult airway of unknown cause. In these patients, it was shown that video laryngoscopy enables visualization of periglottic structures and pathology. Whereas the CLT cannot differentiate PLE from structural laryngeal lesions or laryngeal spasm, video laryngoscopy or flexible endoscopy might potentially enable identification of the cause of the laryngeal narrowing and thus guide treatment. However, further studies on the added value of video laryngoscopy or flexible endoscopy (or both) in the prediction of PLE and PES as well as the diagnostic approach of laryngeal narrowing are needed before they can be implemented in clinical practice.

## Prevention

Elimination of possible risk factors might prevent PLE and thus decrease the incidence of PLE. Firstly, an adequate-size endotracheal tube should be selected. Generally accepted maximum endotracheal tube sizes are 7.0 mm for women and 8.0 mm for men. However, smaller endotracheal tubes may interfere with endoscopic endotracheal procedures and increase the work of breathing and this should be taken into account during the weaning process. Secondly, the duration of intubation should be minimized since the duration of intubation in patients with PES is consistently increased compared with patients without PES. No data on a potential cutoff length of intubation increasing the risk for PES are available; however, in general, extubation should not be postponed in order to prevent unnecessary prolongation of intubation. The application of noninvasive ventilation (NIV) might facilitate early detubation, although no data on the effect of early detubation combined with NIV on PES have been published. Thirdly, cuff pressures should be measured regularly to prevent formation of pressure ulcers due to high cuff pressure. Although no evidence on the maximum acceptable cuff pressure is available, 25 cm H_2_O is a widely accepted upper limit [38]. Since the use of continuous cuff pressure monitoring is also associated with a decreased incidence of ventilator-associated pneumonia, the use of continuous cuff pressure monitoring should be strongly advised [39].

Several studies have investigated the use of intravenously administered corticosteroids in the prevention of LE (Table 5), and initial studies failed to show a beneficial effect of corticosteroids on PLE [12, 17]. However, in more recent studies, corticosteroids were shown to decrease the incidence of PLE by more than 50 % (Table 5) [11, 16, 26, 30]. This difference is probably caused by the implementation of different treatment algorithms. Whereas in the initial studies extubation was performed 1 hour after the administration of the corticosteroids, later studies implemented different treatment algorithms with at least 4–48 hours between administration of corticosteroids and extubation. Furthermore, in later studies, low-risk patients were identified by the CLT and excluded from the study, resulting in a study group with a higher risk for PLE. In a recent trial, corticosteroids (budesonide) were nebulized following extubation and compared with nebulized saline, showing more than 50 % reduction in the incidence of respiratory distress and need for reintubation [29]. However, it should be noted that, although a CLT was performed, low-risk patients were not excluded on the basis of the outcome of the CLT.

**Table 5:** The effect of corticosteroids on postextubation laryngeal edema, stridor, respiratory distress, and reintubation

Author	Year	Intervention	Time before extubation	Intervention group			Control group			
				Outcome parameter	Number	Percentage	Outcome parameter	Number	Percentage	*P* value
Darmon et al. [12]	1992	Dexamethasone 8 mg i.v.	1 hour	Laryngeal edema	11/327	3.4 %	Laryngeal edema	17/337	5.0 %	ns
Ho et al. [17]	1996	Hydrocortisone 100 mg i.v.	1 hour	Laryngeal edema	7/39	17.9 %	Laryngeal edema	10/38	26.3 %	ns
Cheng et al. [16]	2006	Methylprednisolone 40 mg i.v.	24 hours	Stridor	5/43	11.6 %	Stridor	13/43	30.2 %	0.15
				Reintubation	3/43	6.9 %	Reintubation	11/43	25.6 %	NA
		Methylprednisolone 4 × 40 mg i.v.	24 hours	Stridor	3/42	7.1 %	Stridor	13/43	30.2 %	0.005
				Reintubation	7/42	16.7 %	Reintubation	11/43	25.6 %	NA
François et al. [11]	2007	Methylprednisolone 3 × 20 mg i.v.	12 hours	Laryngeal edema	11/355	3.1 %	Laryngeal edema	76/343	22. %	<0.01
				Reintubation	13/355	3.7 %	Reintubation	36/343	10.5 %	0.02
Lee et al. [30]^a^	2007	Dexamethasone 4 × 5 mg i.v.	48 hours	Stridor	4/40	10 %	Stridor	11/40	27.5 %	0.037
				Reintubation	1/40	2.5 %	Reintubation	2/40	5.0 %	0.56
Cheng et al. [26]^b^	2011	Methylprednisolone 40 mg i.v.	4 hours	Stridor	6/38	15.8 %	Stridor	13/33	39.4 %	0.025
				Reintubation	3/38	7.9 %	Reintubation	10/33	30.3 %	NA
Abbasi et al. [29]	2014	Budesonide 4 × 1 mg nebulized	48 hours	Respiratory distress	3/35	8.6 %	Respiratory distress	11/35	31.4 %	0.017
				Stridor	3/35	8.6 %	Stridor	7/35	20 %	0.17
				Reintubation	3/35	8.6 %	Reintubation	11/35	31.4 %	0.017

*i.v.* intravenous, *ns* not significant, *NA* not applicable

^a^Only patients with cuff leak volume (CLV) <110 ml were included. In patients with CLV ≥110 ml, stridor was found in 4.9 % of patients, and 1.4 % of patients were reintubated

^b^Only patients with cuff leak percentage (CLP) <24 % were included. In patients with CLP ≥24 %, stridor was found in 3.8 % of patients, and intubation was performed in 5 % of patients

From these studies, it can be concluded that intravenously administered corticosteroids are effective in the prevention of PLE if started several hours before extubation. This conclusion was supported by two meta-analyses, stating that data from the latest well-designed studies suggest that the incidence of PLE and associated PRF is reduced if intravenously administered corticosteroids are started 12–24 hours before extubation and if multiple doses are administered [40, 41]. However, it should be noted that in the most recent trial corticosteroids were started only 4 hours before extubation and a similar reduction of PLE and reintubation rate was observed [26]. Furthermore, recent data suggest that administration of nebulized corticosteroids following extubation might well be as effective as intravenously administered corticosteroids [29]. Therefore, further research is needed to identify the optimal route of administration and treatment algorithm. Perhaps more importantly, future research should focus on the identification of patients at risk for PLE and associated PRF, enabling targeted treatment of these patients and prevention of unnecessary treatment of low-risk patients.

## Treatment

Reintubation should be performed without delay in the presence of respiratory insufficiency due to PLE. Although NIV has been shown to be effective in the prevention of intubation in respiratory insufficiency in general, it was shown to be ineffective in the treatment of respiratory insufficiency following extubation [42]. More than that, NIV for PRF has been associated with increased mortality, probably due to the increased delay to intubation [43]. Therefore, the use of NIV for PRF failure cannot be recommended.

The use of airway exchange catheters (AECs), which has been included in the Difficult Airway Society extubation guidelines for patients at risk for PRF, provides important advantages [44]. Firstly, the AEC may facilitate over-the-wire insertion of an endotracheal tube in the instance of difficult visualization of the glottis and this is not uncommon in the presence of LE [45]. Secondly, the development of the Ventrain® (Ventinova Medical B.V., Eindhoven) device has made emergency ventilation through a narrow-bore catheter possible [46]. Unfortunately, identification of patients at risk for PRF is difficult since no reliable test is available.

The current treatment of choice for PLE consists of intravenous corticosteroids and nebulized epinephrine. Corticosteroids decrease LE by diminishing the inflammatory response and decreasing capillary vessel dilation and permeability. However, the efficacy of corticosteroids in the treatment of PLE has not been investigated and thus no data on the most effective dose are available. Based on the dosages used in the prevention of PLE, a dosage of methylprednisolone 20–40 mg or dexamethasone 5 mg could be suggested and therapy might be continued for 24–48 hours after extubation [11, 16, 26, 30].

Nebulized epinephrine is thought to decrease LE through vasoconstriction, although high-quality evidence on the efficacy is lacking. In pediatric upper airway obstruction caused by severe viral croup, it has been shown to decrease upper airway obstruction scores, and a beneficial effect has been suggested in upper airway obstruction of other etiologies [47–49]. The optimal dosage is currently unknown, although 1 mg in 5 ml has been suggested [49].

The efficacy of combined intravenous corticosteroids and nebulized epinephrine on PLE has been investigated in pediatric patients, although administration of corticosteroids was started before extubation and treatment was not limited to symptomatic patients [50]. In this overall well-designed study, dexamethasone and nebulized epinephrine did not prevent clinical progression of airway obstruction due to PLE. Therefore, the efficacy of both intravenous corticosteroids and nebulized epinephrine as well as the combination of both treatments has not been established.

The inhalation of a helium-oxygen mixture (heliox) decreases airway resistance and thus work of breathing. Although no evidence on the efficacy of heliox administration in adult PLE is available, a 38 % reduction in respiratory distress score was reported in children with PES, although no change in outcome was found [51]. Therefore, heliox does not lead to a reduction of LE but may decrease work of breathing and buy time to establish a definitive solution for the upper airway obstruction and this may be useful in circumstances in which it is difficult to intubate patients. However, in the context of PLE, it should (similar to NIV) not lead to a delay to intubation since that may potentially lead to a worse outcome.

**Fig. 1:**
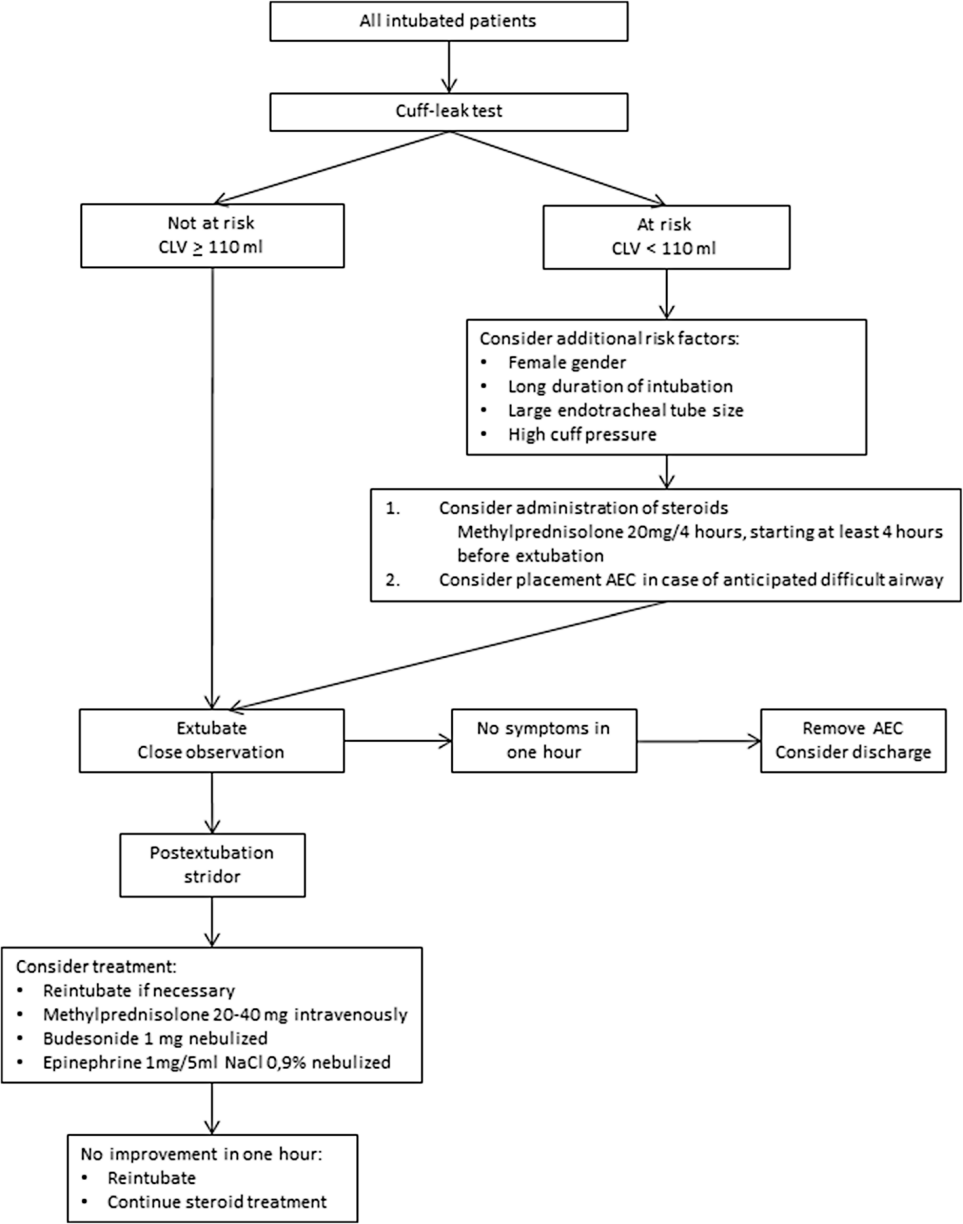
Proposed extubation algorithm. *AEC* airway exchange catheter, *CLV* cuff leak volume. Adapted from Wittekamp et al. [4]

Based on the best available evidence, a practical extubation algorithm, including prevention and treatment of PLE and PRF, may be proposed (Fig. 1). However, it should be emphasized that the CLT effectively identifies low-risk patients only. Treatment of all patients with a positive CLT would result in overtreatment. Therefore, one could decide to perform a CLT in all patients and start pretreatment with corticosteroids in the presence of additional risk factors only, as suggested in the algorithm. Alternatively, a CLT could be performed in patients with risk factors only.

## Conclusions

PLE is a frequent complication of intubation and leads to reintubation in up to 10 % of all extubated patients. Pretreatment with intravenous corticosteroids or administration of nebulized corticosteroids following extubation seems fairly effective in the prevention of PLE, decreasing the need for reintubation by more than 50 %. However, the lack of reliable predictors prevents identification of patients at high risk for PLE and PRF. Patients with a low risk of PLE and PRF can be identified by using the CLT, which is therefore advisable. In patients with a positive CLT, the decision to start pretreatment with corticosteroids should be made on an individual basis and on the basis of the presence of additional risk factors. If corticosteroid therapy is considered to be indicated, treatment should be started at least 4 hours before extubation and multiple doses should be administered. If PES or PLE is present, the treatment of choice should be corticosteroids combined with nebulized epinephrine. However, if PLE or PES leads to respiratory insufficiency, reintubation is the only definitive resolution and should not be postponed.

## Abbreviations

ACW: Air column width
ACWD: Air column width difference
AEC: Airway exchange catheter
CLT: Cuff leak test
CLV: Cuff leak volume
LE: Laryngeal edema
NIV: Noninvasive ventilation
PES: Postextubation stridor
PLE: Postextubation laryngeal edema
PRF: Postextubation respiratory failure
